# Presentation of a new method based on modern multivariate approaches for big data replication in distributed environments

**DOI:** 10.1371/journal.pone.0254210

**Published:** 2021-07-09

**Authors:** Khatereh Sabaghian, Keyhan Khamforoosh, Abdolbaghi Ghaderzadeh

**Affiliations:** Department of Computer Engineering, Sanandaj Branch, Islamic Azad University, Sanandaj, Iran; Gonbad Kavous University, ISLAMIC REPUBLIC OF IRAN

## Abstract

As the amounts of data and use of distributed systems for data storage and processing have increased, reducing the number of replications has turned into a crucial requirement in these systems, which has been addressed by plenty of research. In this paper, an algorithm has been proposed to reduce the number of replications in big data transfer and, eventually to lower the traffic load over the grid by classifying data efficiently and optimally based on the sent data types and using VIKOR as a method of multivariate decision-making for ranking replication sites. Considering different variables, the VIKOR method makes it possible to take all the parameters effective in the assessment of site ranks into account. According to the results and evaluations, the proposed method has exhibited an improvement by about thirty percent in average over the LRU, LFU, BHR, and Without Rep. algorithms. Furthermore, it has improved the existing multivariate methods through different approaches to replication by thirty percent, as it considers effective parameters such as time, the number of replications, and replication site, causing replication to occur when it can make an improvement in terms of access.

## 1- Introduction

Big data replication is a highly significant issue in grid and data environments. It can bring about high efficiency to adopt an appropriate policy for replication of big data in these environments. The purpose is to run an algorithm for big data replication that provides higher efficiency with low computational complexity.

The algorithm for big data replication needs to answer a key question on which big data should be replicated, and where the replication should be placed. Given grid users’ dynamic behavior, it is difficult to decide on data replication to achieve the objective of maximizing accessibility [1]. Replication techniques are categorized into two types: static replication and dynamic replication. In the static replication approach, the numbers of replications and host nodes are specified statically at the beginning of the duty cycle, and there will be no generation or connection of replications or displacement afterwards [2]. Dynamic strategies are compatible with changes in the user’s request pattern, storage capacity, and bandwidth, and can generate replications at new nodes or remove those that are no longer needed based on the general information on the big data grid [2].

Based on a comparison of replication methods considering one or more parameters as the criterion, it can be stated that those using one criterion for replication cause an improvement from one aspect while reducing algorithm efficiency from others. For example, a shorter replication time may be obtained in the algorithm, *i*.*e*., an improvement in the speed parameter, while there are worse results with the criterion of utilized data amount. In algorithms using several criteria, therefore, optimal replication is performed, which can reduce both the amount of data replicated at different nodes and access time. A topic that has recently become highly frequent involves the multi-criteria decision-making model. In such decision-making, several *indices* or *objectives*, sometimes contradictory ones, are taken into account. Multi-criteria decision-making can be categorized as multi-index or multi-objective. Multi-index decision-making models and techniques are used for the selection of the most appropriate from among the *m* alternatives available. Multi-objective decision-making models, on the other hand, consider several objectives at the same time for optimality. The assessment scale for one objective may vary by the parameters considered for the rest. One objective, for example, may be to maximize profit, assessed in terms of money, while another is to minimize the time of employing the workforce, assessed in hours. These objectives are sometimes not in the same direction, and function contradictorily. For instance, the decision-maker may be willing to raise staff satisfaction on the one hand and seek to minimize salary costs on the other.

The Vise Kriterijumska Optimizajica Kompromisno Resenje (VIKOR) method involves a model widely applied in decision-making and selection of the best alternative. The model has been in the process of generation since 1984 based on the consensus method with contradictory criteria and is used in general for solving discrete problems. The method has been developed for multi-criteria optimization of complex systems. It is focused on classification and selection from among a set of alternatives and specifies the compromise solutions to a problem with contradictory criteria, which may assist decision-makers in reaching a final decision. A compromise is a mutual agreement, and a compromise solution is the closest justified solution to the ideal one here.

VIKOR is a ranking method for the solution of discrete problems that considers the importance of distances, unlike other methods such as the Technique for Order of Preference by Similarity to Ideal Solution (TOPSIS). The main difference between this model and hierarchical or network decision models is that pairwise comparison between alternatives based on certain criteria does not take place here. Each alternative is measured and evaluated independently by a criterion that can be more efficient depending on the problem. In this model, there are always several different alternatives that are evaluated independently based on several criteria and finally ranked in terms of value, agreed solutions, and conflicting criteria. Methods have already been presented that may solve the problem with more accurate, better results, but this paper aims is to improve the results of addressing big data as compared to small-scale data, and the VIKOR method is used only as an example problem-solving tool.

The parameters effective on replication include the request for data to be replicated, a factor that allows replication to be performed effectively and conditionally upon requirement. Requested data amount is another highly effective parameter; the smaller the amount of data to be replicated, the lower the traffic load imposed on the grid. It can be stated that excessive data replication and storage space loss can be prevented through more accurate replication, while data will be replicated faster since less traffic is imposed on the grid. This is achieved by the method proposed based on VIKOR, specifying which data should be replicated and where should occur by considering specific parameters such as the number of replications and the amount of requested data. Therefore, it can be stated that the optimal method should be capable of performing a replication that minimizes data access time for requesters with the least space taken up and the lowest traffic load imposed on the grid in the shortest time.

In the method proposed in this paper, the environment for distribution and replication is first established, and the node sites are specified. Then, nodes requesting data send their requests to be received by nodes containing big data. If there are data in the absence of replication, therefore, traffic load will dramatically increase, and then all routes involving the requested data will suffer heavy grid traffic. That is why attempts are made to adopt a way of managing big data replication efficiently using the proposed method to reduce the resulting traffic load over the grid. Nodes with the same or relevant big data are categorized in the same group, and finally, send the same data in a replicated class. The obtained class can be large and contain a large number of nodes. Thus, the eligibility of nodes with big data in a class needs should be verified by an algorithm in terms of distance, the number of user requests, and other parameters before they can help select sites with priority for data replication. That is, big data are replicated only at nodes with advantages over others, which can achieve their requested big data in a shorter time.

The proposed algorithm is innovative as it

1. uses different criteria in data replication
2. prioritizes the parameters by assigning a different weight to each
3. reduces replication time and data amount given the importance of the data replication type so that a smaller amount of data is replicated, and total replication time decreases.

This paper is presented in five sections. An introduction is provided in Section 1. Section 2 contains a review of the literature and an examination of the pros and cons of earlier works, the proposed algorithm is presented in Section 3, Section 4 provides an evaluation, and a conclusion is made in Section 5.

## 2- Review of the literature

A huge number of algorithms have been proposed for big data replication, each with its pros and cons. The problem of replication is an optimization problem to which solutions have been presented. The optimization can involve a search for the best host for files of different sizes. Some of the algorithms perform the replication based on data mining and identification of relevant data to be placed at adjacent nodes, and others find the best data for the best sites as optimization problems based on univariate and multivariate solutions. Given the high complexity of this type of problem, there are also papers using metaheuristic solutions and the relevant methods. In an algorithm presented as prediction-based modified BHR (PMBHR), data mining techniques are used for the prediction of access to data in the future. Based on the appropriate sites selected for replication and of the prefetched required files, the method provides local access and reduces costs over the grid [3].

In another research, replicas provide an efficient way of confronting the challenges involved in the improvement of data management over a big data grid. Different strategies are presented for replication, which is aimed mainly at raising performance in data grids. In this case, knowledge of files can be extracted from historical and operational data using data mining techniques. Data mining techniques provide a powerful tool for facilitating the extraction of meaningful knowledge from a big data set. In this paper, the extracted knowledge is used for the promotion of replica site management [2]. Moreover, the data replication algorithm is presented for a group of files over a data grid known as Popular Groups of Files Replication (PGFR). In this algorithm, file users in a connected graph are categorized into groups with similar interests to identify a group of dependent files and add them to the grid. This increases the capability of gaining local access. The evaluation of the results demonstrates that the presented algorithm performs better, and minimizes mean task runtime, bandwidth consumption, and unnecessary replication. Replication will not be performed in the algorithm if a large amount of data is sent, and grid bandwidth is low. In other words, the different aspects of replication are considered in each case before it can be performed [4]. A dynamic replication management algorithm on distributed data is presented in [5], which resolves the response time issued by providing the relevant data to prevent delayed reading of information from the disks. In this paper, a new method is presented for the performance of replications given the selected set and number of replications, their techniques of placement, and the calculated popularity of the data in terms of their access history and timeliness. In [6], the replication set is selected based on recent popularity, and the number of replications for each replica is suggested. Finally, the algorithm places the replications at the appropriate sites to balance the load. In [7], a supercomputer is used for large amounts of calculation in scientific applications in charge of processing big data, and distributed data centers around the world are used as resources. A system model is presented for cooperation from different data centers and for the satisfaction of client requests and identification of data centers to provide the client with the shortest response time. Moreover, the dynamic data replication strategy at distributed data centers is accurately examined in terms of popularity. It is demonstrated in [8] that the information grid is an integrated architecture including several computers, where the resources are connected in distributed environments. Data file replication is an effective capability in grids that both minimizes data access time and improves total access by replicating data at the most easily accessible appropriate site. The performance of a replica or data replication depends on different factors such as the selected replication site, method of placement, and grid traffic and bandwidth. In [1, 2, 9], grid calculations are made all around the world for the provision of huge computational power and extensive distributed storage. A problem with data access over data grids involves the selection of the appropriate site for replica placement. Genetic Algorithm (GA) and Simulated Annealing (SA) are two popular evolutionary algorithms inspired by the nature. In this paper, a hybrid method is known as Hybrid Genetic Algorithm and Simulated Annealing (HGASA) is proposed to solve the problem of selecting the replica site in the data grid. The proposed algorithm raises grid performance by improving security, file accessibility, load balance, and response time.

In [10, 11], a data replication strategy, referred to as RSPC, is proposed where systems are rented for replication and profit and cost calculation at data centers. It is highly challenging to pay for the data replication system rent and ensure economic profit for the cloud providers at the same time, as discussed in that paper. Moreover, a hybrid method based on voting structures is presented for replication of new hybrid data. On that basis, data with the greatest capability of replication gain higher scores, which advantages them in the replication procedure [12]. Data replication is used as an efficient method that reduces retrieval time and, consequently, energy consumption in the cloud [13]. If the required files are inaccessible locally, they will be received from places far away, which is highly time-consuming. To address this issue, a new dynamic replication strategy, referred to as Prefetching-aware Replication Data (PRD), is presented that specifies the correlations between data files using the history of access to them and receives the most popular in advance. Thus, the file will be accessible locally the next time it is needed. Four input parameters are used in the paper based on the fuzzy inference system: the number of accesses, cost of replication, last access to the replicated data, and data accessibility.

The parallelization technique is used to split large work streams into several small workflows. It also utilizes the data replication algorithm for run-time access to the data [14–16]. The major characteristics of workflows play an important role here, including runtime, dependency patterns, and file size, with the latter two parameters used for running four workflows. The obtained results confirm that the dependency patterns of the workflow significantly affect the performance of the scheduling strategy. A new dynamic adaptive replica strategy (HGASA) is presented in [17] to minimize the number of node overheads. The strategy performs replications given the great similarity to the specification of the membership function based on node load. If there is extreme similarity, a new replication will be generated. The fuzzy clustering strategy is then applied for the placement of replications, stored in the node with the highest degree and least similarity. This strategy can reduce the number of high-load nodes and data access time. [18] introduces a new dynamic replication strategy given file heat and site load (HGASA) to prevent unnecessary replication.

The job proposed using multi-dimensional parameters is used for the prediction of the future. Simulated in the OptorSim optimizer, the proposed replica replacement algorithm utilizes normalization and weight values for the prediction of its subsequent uses. Contact rate, mean job runtime, efficient use of the grid, and calculation elements are used for the evaluation of the algorithm performance [19]. A recently developed method of decision-making, Combined Compromise Solution (CoCoSo) integrates Simple Additive Weighting (SAW) and Exponentially Weighted Product (MEW). It deduces compromise solutions to MCDM problems through the combination of the compromise decision-making algorithm and aggregation strategies. Despite the reliability and stability of the decisions, it is not affected easily by changes in the weight distribution of criteria [20]. The results obtained by the Weighted Sum Model and the Weighted Product Model are combined in WASPAS. The values of the combined optimality criteria determine the list of alternatives. The method is capable of maintaining consistency in alternative rankings; however, through sensitivity analyses [21]. Introduced in [22], the EDAS (Evaluation based on Distance from Average Solution) method is applied to the problem of inventory classification, and its efficiency in addressing MCDM problems is examined. It specifies how desirable an alternative is according to its positive and negative distances from a reference solution known as the average solution, assigning greater desirability to alternatives with higher values of positive distance and lower values of negative distance from the average solution.

A new multi-objective non-linear programming model is formulated in [23] for simultaneous evaluation of criteria and alternatives (SECA) in MCDM problems. The proposed method is based on the maximization of the overall performance of alternatives given the decision matrix information on variation within and between criteria measured using standard deviation and correlation, respectively. The analysis results indicate the efficiency of the model in addressing MCDM problems. A novel idea is used in MEREC (Method based on the Removal Effects of Criteria) for weighting criteria. Its efficiency is confirmed through computational analysis following its systematic introduction. [24] involves an illustration of the MEREC procedure for criterion weighting, along with a comparison using an example for the validation of the results of the presented method. A simulation-based analysis is also made for the verification of the reliability of the procedure and stability of its results.

A technique for prioritization of replication sites is presented in [25]. In this method, similar replications are located far apart, and different ones are close to each other. The shortest path between the two sites is selected using Dijkstra’s algorithm, decreasing access time and increasing efficiency. Another paper lists six different replication strategies in the access to a tree: 1- no replication, where only the root is replicated, 2- best client, where replication is performed for the client with the greatest access to the file, 3- cascade, where replication is performed on the best service receptor’s route, 4- plain cache, where local replication is stored based on the first request, 5- cascade cache, a combination of the above two strategies, and 6- fast spread, where the files are stored on the route taken by the best client at each replication node, and response time and consumed bandwidth are assessed. The latter technique increases accessibility and decreases response time and consumed bandwidth, but does not increase load balance [26, 27]. Table 1 shows the summary of these important algorithms.

**Table 1:** A summary of the reviewed methods.

Ref	Author(s)	Year of publication	Algorithm used in the paper	Description of the utilized method
[3]	Beyg Rezayi et al.	2014	Replication based on data mining	The method works through the prediction and estimation of the probability of replication.
[4]	Ghilavizadeh et al.	2013	Group data replication	The method uses a graph of similar data to reduce runtime and consumed bandwidth.
[5]	Azari et al.	2018	Distributed dynamic replication	The method works based on data popularity to specify where replication should be performed.
[7]	Xiong et al.	2018	Big data replication	Data are replicated based on the requests in a distributed architecture.
[8]	Jayalakshmi et al.	2016	Distributed data replication	Data are replicated based on parameters such as replication site, method of placement, and grid traffic, with the obtained results capable of increasing efficiency.
[1]	Grace et al.	2016	Combination of genetic and evolutionary algorithms in data replication	The presented algorithm can decrease traffic load and response time and increase load balance in the grid using a hybrid method.
[10]	He et al.	2020	Profit-based replication	Replication is performed based on profit, cost, and economic estimation.
[12]	Eid et al.	2020	Hybrid data replication	Replication is performed based on data score.
[13]	Bokhari et al.	2020	Replication based on the fuzzy method	Replication time, the number of replications, replication cost, *etc*. are reduced based on dynamicity, applied to the algorithm as specified by fuzzy methods.
[14]	Mansouri et al.	2018	Replication based on parallel methods	Parallel methods are used to reduce data access time, and a workflow method is obtained through parallelization of jobs.
[17]	Casas et al.	2017	Fuzzy cluster replication	The method uses fuzzy clustering, which allows data replication without extreme load imposed on the grid, and reduces access time.
[18]	Sun et al.	2018	Dynamic replication	Dynamic data mining methods are used for reduction of unnecessary replications, which increases grid load.
[19]	Muthu et al.	2019	Multidimensional replication	Multidimensional methods of data replication are used, which reduces mean runtime in the loading of replicated data.
[25]	Challah et al.	2010	Minimum graph algorithm	Methods of obtaining shortest paths, such as Dijkstra’s algorithmare used, which can decrease access time and increase efficiency.
[27]	Ranganathan et al.	2001	Fast data replication	several methods are presented, which can significantly reduce access time through appropriate placement by replicating the best rather than all data.

## 3- Motivation

The proposed method has a number of advantages, which can be used to resolve many issues, including the employment of four important, basic criteria in evaluation, unlike in other articles, where fewer criteria are used for that purpose. This provides better, more accurate results. Moreover, the nodes are prioritized through adoption of an efficient weight assignment strategy based on the VIKOR method, which leads to the high accuracy of the final results. Total replication time and data volume are reduced given the significance of data replication. Through application of the VIKOR method rather than simpler methods such as TOPSIS, the importance of distances other than those to the best and worst nodes is considered, which leads to better, more accurate results. However, given that the aim is to compare performance on small-scale and big data, the improvement made for big data eliminates the need for more sophisticated methods such as CoCoSo, WASPAS, SECA, and MREC, although application of these methods may also provide more accurate results.

## 4- Proposed method

**Fig 1:**
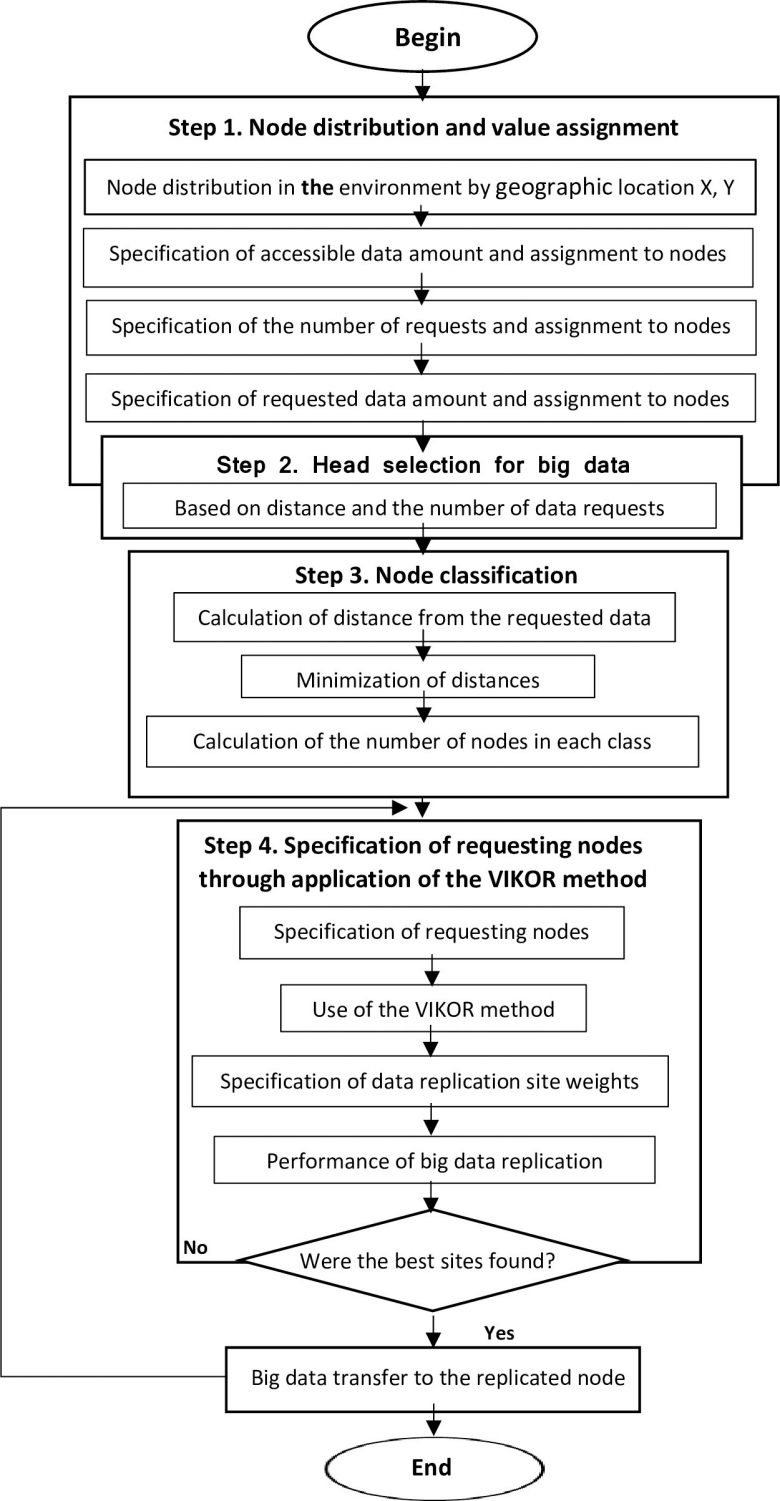
Flowchart of the proposed method.

The purpose of the proposed method is to offer proper performance in big data replication by utilizing multivariate selection algorithms. In the proposed method, Replication VIKOR (RV), the problem inputs include node distribution, number of nodes, data amount for replication, and number of requests, and the final problem outputs include replicated data space, best site specification for replication, and the use of the VIKOR method for ranking and prioritizing the nodes selected for replication. Each of these data is placed in a group of nodes close together so as to minimize distance. If big data replication is performed improperly in the group, therefore, traffic load over the grid will increase upon request for big data from a node outside the group, and large amounts of data will be sent far away in the grid. If the grouping is carried out properly. However, traffic load over the grid can decrease significantly, and the appropriate site for replication among the data and nodes can be identified using the proposed method, so that replication is performed only at eligible nodes. The flowchart in Fig 1 provides an overview of the proposed method in four steps:

### Step 1. Node distribution and value assignment

In the first step of the proposed method, the nodes are randomly distributed in environments of different sizes. Each of the nodes distributed in an environment range has its own features. Each node is randomly assigned a data amount from 1 to 50 gigabytes, and the other nodes randomly request the assigned data amount range from 1 to 40. In this scenario, the data in a certain amount assigned to different nodes are requested from the other nodes in different numbers. If a node requests 40 gigabytes of data, for instance, it will need to receive the entire data as one from a single node, and the data will be sent when the size equals the requested amount. In other words, a single data package is sent in this scenario.

### Step 2. Head selection for big data

Once the requested big data are specified among the distributed nodes in the second step, each group needs a leading node as the head node, transferring the information collected in each group to the others. Heads are selected from the margin, coordinates between 35 and 65 for the *X*s and *Y*s, with a threshold of 20 percent of the total number of distributed nodes. Then, requests are collected from all the nodes, and the data requested from other nodes are located. The operation is carried out through broadcasting, after which each node realizes what data are contained at which nodes, and where each other node is located. Then, its distances to nodes possessing the data are calculated given the *x* and *y* values of each node, along with the size and amount of requested data, and then their candidacy as candidate replication sites can be examined.

### Step 3. Node classification

The nodes are classified in the third step, where the packet type sent from each class is specified, indicating the data types requested by different nodes for sending, from which an appropriate node is selected for replication. Big data that are physically close to each other and the same requests are grouped in the same class. For that purpose, the distances from each node to the specified heads of all the big data classes are first calculated and then sorted in ascending order. Thus, data can be added to a class when located at the shortest distance to the head, and there is a counter to specify the number of nodes in each class. Finally, the index of the shortest distance from the requested node is stored in a variable. The classification demonstrates that a class is optimal if it involves only one replication from each type of big data. On that basis, this operation can prevent irregular replication to obtain appropriate data with efficient outputs for replication. It should therefore be noted that classification should be made accurately, as a node excluded from a class containing several may have a request for specific data.

### Step 4. Specification of requesting nodes through the application of the VIKOR method

The fourth step involves a specification of requesting nodes, depending on their amounts of data request from a specific dataset and stored in a matrix with elements including all the nodes available in each class except the one to which the requested node belongs. Thus, if the requested big data are so large that replication is not optimal for them, or the big data are requested by only one node, the node cannot be regarded as a specific data requester appropriate to the operation of big data replication. The big data requester sends the request to a specific node, but it depends on the VIKOR evaluation from which replica the big data are sent.

In the proposed method, ranking is carried out for the requested data amount, the number of requests for replication data, and the free space for the storage of each node in the distributed environment, which is why it can be used to specify the fitness values of candidate nodes for replication.

In the VIKOR method, a matrix of replicated data is considered as input, along with sites ranked for replication taken as output. The output sites are eligible for big data replication based on the VIKOR index. In other words, VIKOR specifies the eligibility of each node. First, a coefficient of 0.5 is considered, which means that the values of mean and maximum distance to the ideal point are equal. On that basis, the decision matrix of the data is drawn, where each row represents a node, and the columns are set based on accessible data amount, requested data amount, the number of requests for data, and distance. Given the weights assigned to the above parameters, the ideal and anti-ideal nodes are obtained, along with the distances between each node and the above two. Then, the criteria of maximum utility and minimum regret are calculated for the node, obtaining the VIKOR index, which indicates the value of each node. The nodes can be considered as ranked by the VIKOR index when numbers closer to one represent greater priority and those closer to zero signify less priority, and all the sites eligible for big data replication are ranked by node fitness. Every time big data is replicated, all the above steps are taken once again, so that the nodes can be reconsidered in the new ranking. The VIKOR method simply provides some prioritization among the nodes, with no changes made in any of the heads. The classes represent common requests; therefore, VIKOR only ranks the replication sites in each class and among all the classes at the final point. Thus, nodes are always selected for replication that can actually reduce data transfer and traffic load over the grid.

The steps of the VIKOR method are as follows.

1. In the first step, the decision matrix is created according to Eq 1.
$$X=\left[\begin{array}{ccc} x11 & x12\cdots & x1j\\ \vdots & \ddots & \vdots \\ xi1 & \cdots & xij \end{array}\right]$$ (1)
  In (1), x_ij_ is the value of record *i* with respect to criterion j. In this paper, the decision matrix is a 20 x 4 matrix, where the rows contain the numbers of the data centers hosting the files, and the columns involve distance, time, percentage of occupied space, and number of requests.
2. A standard matrix is formed from the decision matrix generated in step 1 through normalization of the data seen in Eq (2).
$$R=\left[(\begin{array}{ccc} r11 & \cdots & r1n\\ \vdots & \ddots & \vdots \\ rm1 & \cdots & rmn \end{array})\right]$$ (2)
  In Eq 2, each element is calculated from Eq 3. In Eq 3, xij is the mean of record i with respect to criterion j.
$$r_{ij}=\frac{x_{ij}}{\sqrt{\sum_{i=1}^{m}{x_{ij}}^{2}}}$$ (3)
3. The coefficients of importance of the different criteria in the decision-making are expressed as a weighted vector in Eq 4.
W=[w1,w2,…,wn] (4)
  The decision matrix is weighted through multiplication of the weights by the elements.
4. The best values for the positive and negative criteria are calculated from Eq 5. In this relation, f_ij_ represents the value of each criterion in the record in the weighted normal matrix.
$$f_{j}^{*}=maxf_{ij}$$ (5)
5. The worst values for the positive and negative criteria are calculated from Eq 6. In this relation, f_ij_ represents the value of each criterion in the record in the weighted normal matrix.
$$f_{j}^{-}=minf_{ij}$$ (6)
6. The utility (S_i_) and regret (R_i_) values are calculated for each solution.
  - The utility index (S) is obtained from Eq 7.
$$s_{i}=\sum_{i=1}^{n}w_{i}\frac{f_{i}^{*}-f_{ij}}{f_{i}^{*}-f_{i}^{-}}$$ (7)
    In this relation, the alternative f* represents the largest number of normal weight matrices for each column, f_ij_ indicates the desired value for each criterion in the record in the normal weight matrix, and f^-^ denotes the smallest number of normal weight matrices for each column.
  - The regret index (S) is obtained from Eq 7.
$$R_{i}={max}_{i}\;[w_{i}.\frac{f_{i}^{*}-f_{ij}}{f_{i}^{*}-f_{i}^{-}}]$$ (8)
    In this relation, the alternative f* represents the largest number of normal weight matrices for each column, f_ij_ indicates each criterion in the record in the normal weight matrix, and f^-^ denotes the smallest number of normal weight matrices for each column.
7. The VIKOR (Q_j_) index for each solution is calculated. The calculation of Q provides the final values of the records to obtain the final ranking for replication, based on Eq 9.
$$Q_{j}=v.\frac{S_{j}-S^{-}}{S^{*}-S^{-}}+(1-v).\frac{R_{j}-R^{-}}{R^{*}-R^{-}}$$ (9)
  In this relation, V represents a fixed number, 0.5. S_j_ indicates the sum of the values of S, S^-^ denotes the largest index of S, and S* denotes the smallest index of S for each record. R_j_ represents the sum of the values of R, R^-^ indicates the largest index of R, and R* denotes the smallest index of R for each record.
$$S^{-}=mins_{i}$$ (10)
$$S^{*}=maxs_{i}$$ (11)
$$R^{-}=minR_{i}$$ (12)
$$R^{*}=maxR_{i}$$ (13)
  The calculation of the VIKOR index in these equations expresses the distance rate from the ideal and that from the anti-ideal limits, and parameter V is set as agreed by the decision-making group.
8. The solutions are sorted by S_j_, R_j_, and Q_j_. At this stage, the values of R, S, and Q are arranged in three groups from small to large. Finally, an option is selected as the top one if recognized as top in all the three groups.
9. A compromise solution is proposed given the following two conditions.
  - Acceptance advantage, meaning that the compromise solution must be considerably different from the subsequent solution.

If A1 and A2 represent the first and second top options in the group, respectively, and n indicates the number of records, Relation 14 must hold.


$$Q(A_{2})-Q(A_{1})\geq \frac{1}{n-1}$$ (14)

- Acceptable stability in decision-making, meaning that the selected compromise solution must have maximal group utility and minimal individual regret.

If the first condition is not met, a set of top records will be selected as follows, in which case Relation 15 must hold.


$$Q(A_{m})-Q(A_{1})<\frac{1}{n-1}$$ (15)

## 5- Simulation and evaluation

Changes occurring in the method, time, or other parameters affecting the way the data are sent result from the fact that a change in an aspect of the replication environment can decrease the duration of the process of sending data. On the other hand, an increase in the number of head nodes can increase traffic load there even if suboptimal, which may result in low efficiency.

In this paper, the OptorSim software is used for the evaluation of the proposed method, implemented under the same conditions as for the Technique for Order of Preference by Similarity to Ideal Solution (TOPSIS), Simple Additive Weighting (SAW), Without Replication (Without Rep.), Least Recently Used (LRU), Least Frequently Used (LFU), and Bandwidth Hierarchy Replication (BHR) algorithms, and the results are reported. A system with the following specifications is used for that purpose. Table 2 shows hardware parameters.

**Table 2:** System used for simulation.

Parameter	Value
RAM	6 GB
CPU	i5-2430M
HDD	240GB SSD
OS	Windows 10 Pro
Software	OptorSim
Dataset	Default OptorSim

Table 3 examines the parameters used for simulation. As it can be seen, small-sized data are also input to the proposed algorithm so that its performance can be evaluated with them as well as with big data.

**Table 3:** Parameter values considered for the proposed method.

Parameter	Value
Accessible data amount	10MB-50GB
Requested data amount	10MB-50GB
Number of data requests	1–40
Distance	100*100
Node distribution	Random
Number of nodes	40
Space used by each node	10MB-40GB

In this section, the proposed method is compared under the same conditions to Without Rep., involving a mechanism without replication. It distributes the data to be used statically in the system, where no change can be made at run time. This algorithm provides a basic method used for evaluation in almost all papers to help obtain the efficiency of replication. In LRU, which replicates the most recently used data, replication is carried out locally. For that purpose, common space is first considered for each of the data in the local grid. That is, data are stored in memory shared by all the requesting nodes when they need to be replicated. If the data are larger than the common space, the most recent item available in memory used least frequently is removed, and this is repeated until all the data are shared and used by other users. In LFU, the least frequent site is replicated. Unlike the above method, removing only the latest sites used less frequently, this algorithm stores data that need to be replicated in common local memory. If the data need a larger space than common in this method, those used less often are removed after the examination. In BHR, the hierarchical replication of bandwidth and data is performed locally. The strategy of the algorithm involves local grids, where all nodes close together are considered as one since there are fast connections between them, causing replication at the nodes.

### 5-1- Replication examined in large- and small-sized sent files

**Fig 2:**
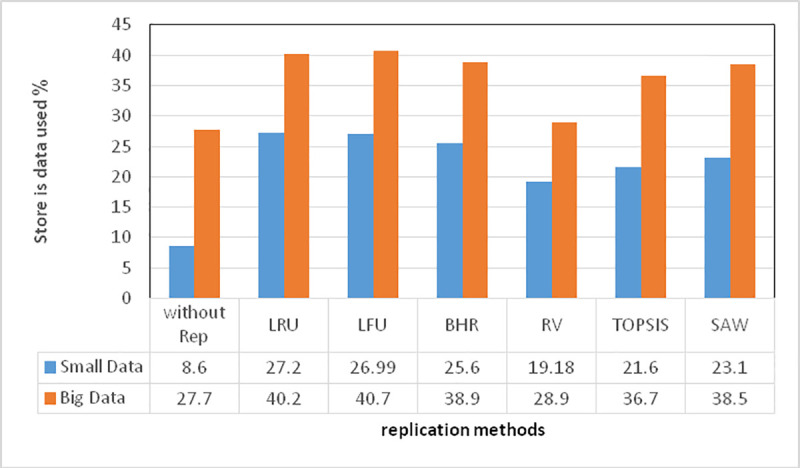
Space taken up by data replication algorithms.

In the implemented scenario shown in Fig 2, the number of selected nodes in all the algorithms is 200, environment size is 200*200, file size is 40GB, the number of requests is 30, and the nodes, of size 40GB each, are randomly distributed over the environment. The sender and a receptor node are then specified, for each of which the requested data amount is considered randomly: between 10 and 40 gigabytes for big data and between 10 and 40 megabytes for small-sized data.

In Fig 2, the percentage of space taken up is represented in gigabytes and megabytes for big and small-sized data. For big data, the proposed algorithm has used less space than the other methods except Without Rep., enabling the nodes to always possess part of the storage space left empty for the performance of the assigned tasks. Although the smallest number of replications concerns Without Rep., capable of taking up less space without replication, the provided service is improper in terms of time due to the lack of replication. In the proposed algorithm; however, the number of requests and the requested amount of data have been considered as criteria along with replication sites to reduce access time. For small-sized data, there have been a larger number of replications more repeatedly, and more data have been replicated. As replication with such data is easier, the little workload is imposed, which causes a larger space to be taken up. The proposed algorithm; however, has offered more proper output also with small-sized data, because all the factors have been considered in data replication. That is, data have been replicated at the best possible point with the highest amount of requested data so that the slightest replication can provide the greatest accessibility.

**Fig 3:**
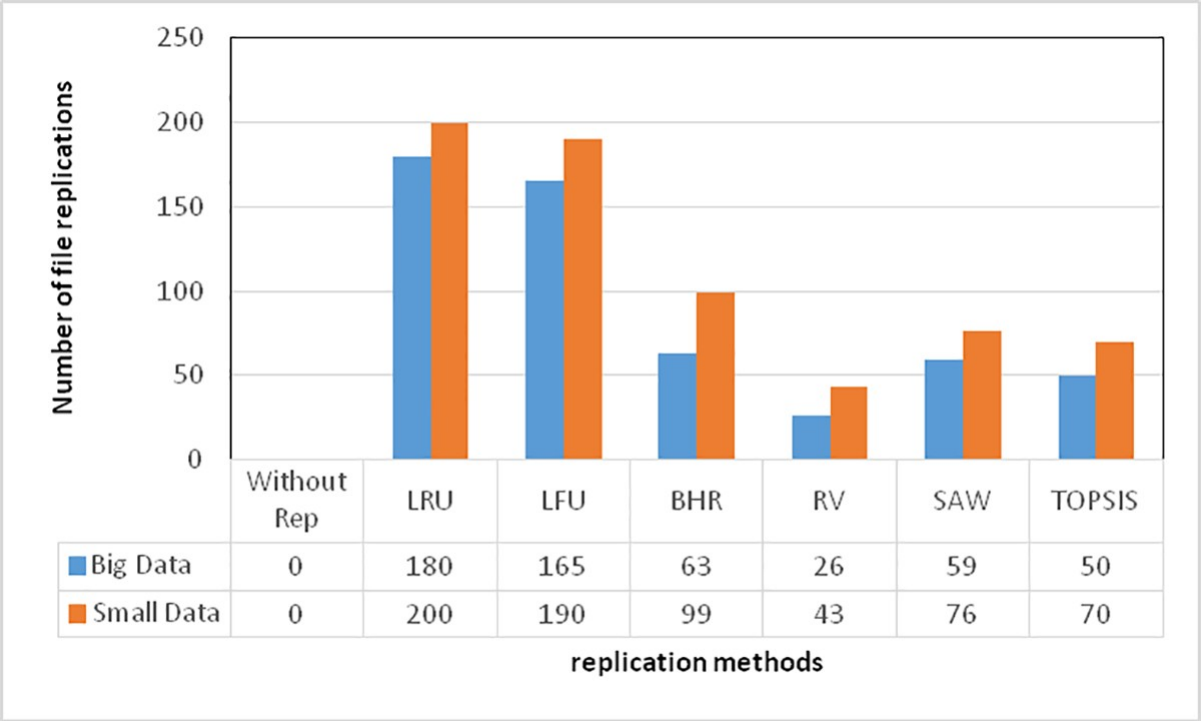
A comparison of the number of replicated files in the examined methods.

In Fig 3, the number of replicated files is considered for both big and small-sized data given the following scenario, and the obtained output is detailed below. The proposed algorithm has been run in a 200*200 environment, where the data have been distributed randomly. Node replication has been performed in two modes of data amount: big and small-sized. Thirty requests have been considered for each data replication, based on which, it can be stated that the proposed algorithm has responded to user requests for replication in big data (40 gigabytes) with a smaller number of replications. For small-sized data (10 megabytes); however, there has been greater replication as little traffic load has been imposed over the grid. It can be found from the following table that the proposed algorithm can also exhibit proper output for small-sized data. Since ranking is carried out once again for each of the requested data, data replication is, however, accompanied by long response times, which can be regarded as a drawback of the algorithm if ranking is carried out in each round of data replication.

### 5-2- Replication examined based on the number of nodes in large- and small-sized files

**Fig 4:**
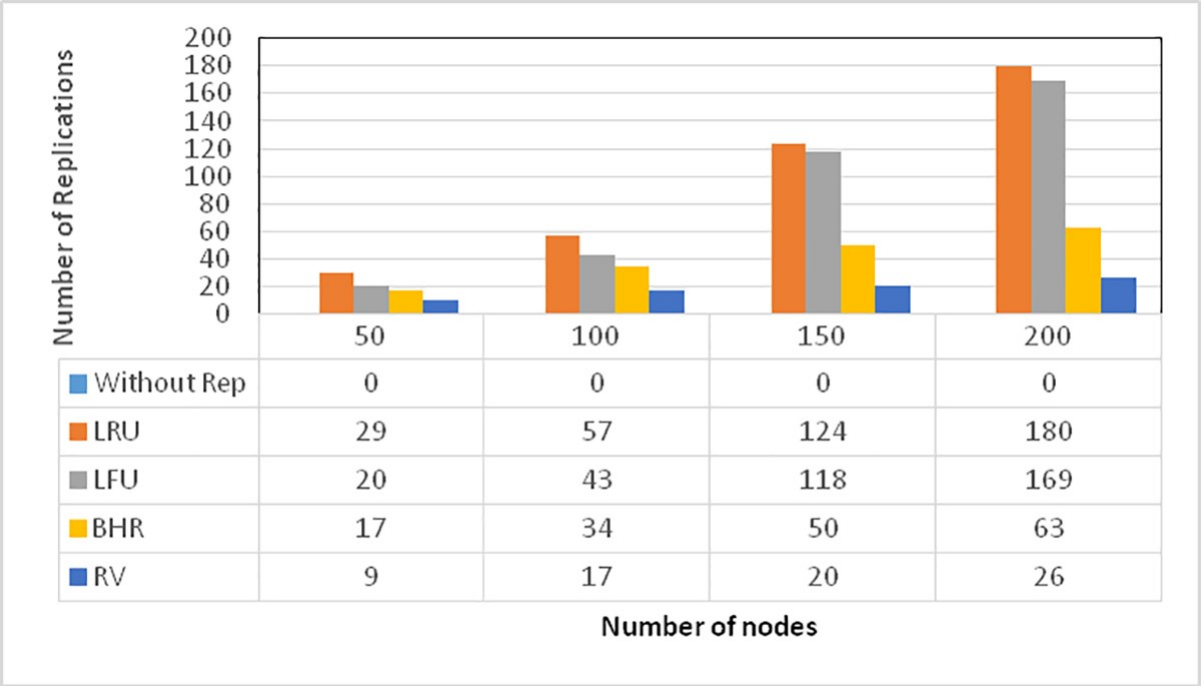
An examination of the number of replications for a variable number of nodes.

This section addresses the number of nodes in a 200*200 environment with data of large and small sizes. As shown in Fig 4, the number of replications is considered in fixed environments with 50, 100, 150, and 200 nodes. The evaluation indicates that the number of replications has decreased more in the proposed algorithm where the number of nodes has increased.

It is observed in Fig 4 that the proposed algorithm has maintained efficiency proportionally to the number of nodes distributed in the environment, indicating that less replication has been performed as the number of nodes has increased. In this examination, there have been a variable number of nodes, randomly distributed in the environment in 50, 100, 150, and 200-node ranges. Capable of providing appropriate service with fewer replications, the proposed algorithm has exhibited the least replication and, eventually, the best output as the number of requesting nodes has increased.

As it can be observed, the proposed algorithm can perform more properly with a larger number of nodes than smaller ones. Of course, it has offered the least replication even in the latter case. This improvement is caused either due to the number of requests or the distance on which the evaluation is based.

### 5-3- Replication examined based on distance in large files

**Fig 5:**
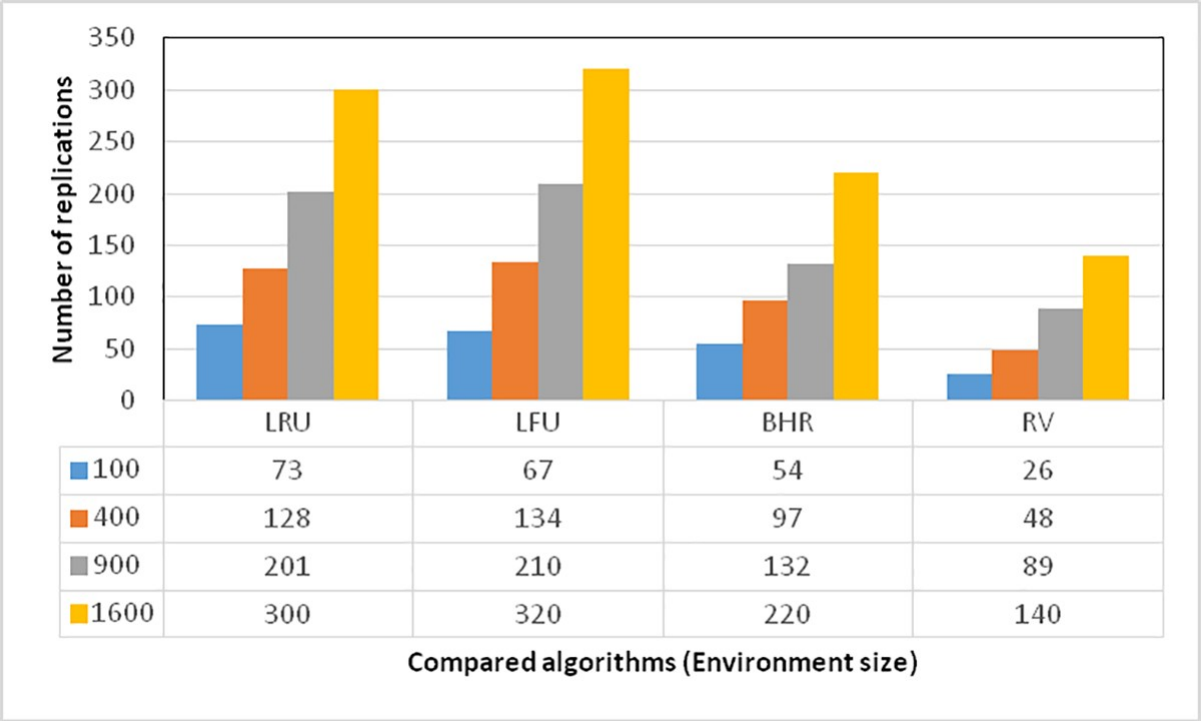
Relationship between the number of replications and replication environment.

A scenario is pursued in this section for the specification of the ratio of environment size to the number of replicated data, enabling an evaluation of the changes in the algorithm with changes in environment size for large files. More specifically, the evaluation involves an examination of the changes in the number of replications in the proposed algorithm and the other three for large files as the area increases from 100*100 to 1600*1600. As it can be seen in Fig 5, the proposed algorithm has performed well for large files in environments with larger areas and exhibited proper performance with the smallest number of replications.

The proposed algorithm provides service in an environment with an area of 100 square meters and fewer replications. This value is about 50 percent that in BHR and 25 percent that in LFU and LRU. Moreover, it remains almost constant as the area increases to 1600 square meters. For the selection of appropriate data replication sites in the comparison between the proposed algorithm and the others, an environment can be selected with the same ratio of access to requesters so that access time is divided between them. In the LRU algorithm, however, data are replicated based on the number of recent requests, and access time does not play a significant role there.

### 5-4- Runtime examined for large and small files

**Fig 6:**
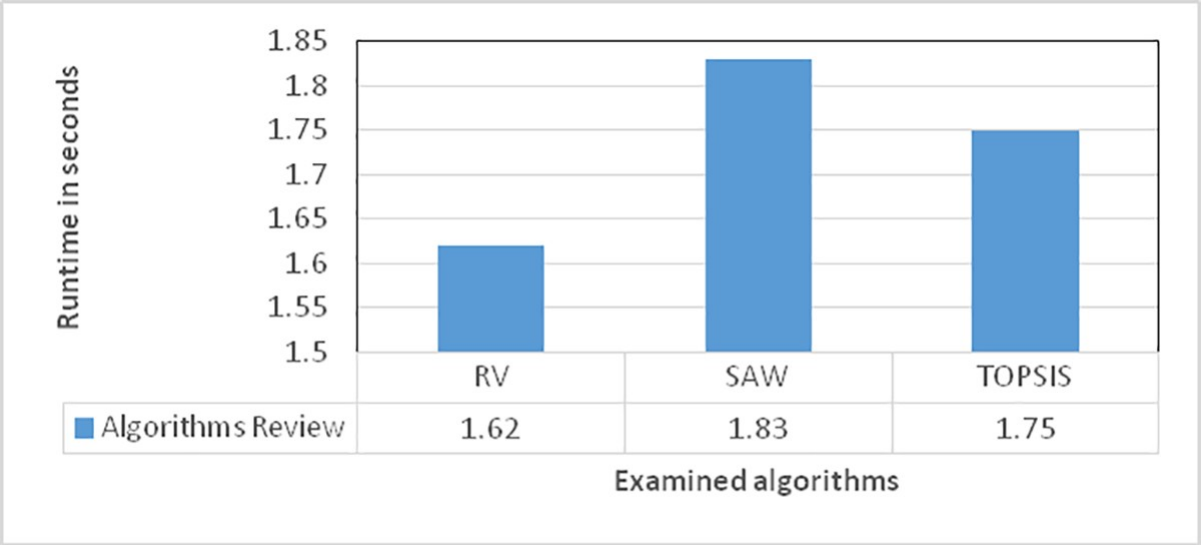
A comparison of the proposed algorithm and the other methods.

In Fig 6, the proposed algorithm is compared to the other multivariate methods in terms of runtime, which demonstrates that the modifications made in the proposed algorithm have reduced its run time, enabling it to run faster. The performed rankings reduce the effects of environmental factors, and a single ranking can specify the optimal site for data replication, not requiring the ranking algorithm to run again in subsequent replications. This makes it possible to specify the sites for more replications with no computational complexity imposed on the grid.

TOPSIS and SAW are the methods involved in the evaluation, where the latter has exhibited close performance to the proposed method, with a similar structure to that of TOPSIS. However, the proposed algorithm reduces data replication in its running procedure by specifying positive and negative parameters, which causes it to run more slowly.

## 6- Conclusion

In this paper, the VIKOR algorithm has been used for obtaining proper results from big data replication, problems involve the traffic load that it imposes on grids and the space used by nodes for consecutive replications. Therefore, attempts have been made to eliminate the above problems from the proposed algorithm and to present a method based on the VIKOR algorithm using multi-criteria decision-making algorithms to reduce big data replication. The proposed method can improve efficiency, as demonstrated by the comparisons made to those involved in big data replication. In future research, the number of parameters effective on replication can be increased to achieve more optimal, more efficient replications. Moreover, other methods of multivariate selection or a combination of two or more methods may obtain more appropriate results.

Furthermore, it is recommended that methods such as CoCoSo, WASPAS, SECA, and MEREC be applied to big data and evaluated and compared to obtain higher accuracy and shorter execution time and that the best methods and best results for replication of big data be introduced.

## Supporting information

S1 Data (XLSX)

S2 Data (XLSX)
